# Association Between Left Ventricular Global Function Index and Outcomes in Patients With Dilated Cardiomyopathy

**DOI:** 10.3389/fcvm.2021.751907

**Published:** 2021-11-16

**Authors:** Tong Liu, Zhen Zhou, Kairui Bo, Yifeng Gao, Hui Wang, Rui Wang, Wei Liu, Sanshuai Chang, Yuanyuan Liu, Yuqing Sun, David Firmin, Guang Yang, Jianzeng Dong, Lei Xu

**Affiliations:** ^1^Department of Cardiology, Beijing Anzhen Hospital, Capital Medical University, Beijing, China; ^2^National Clinical Research Center for Cardiovascular Diseases, Capital Medical University, Beijing, China; ^3^Beijing Advanced Innovation Center for Big Data-Based Precision Medicine for Cardiovascular Diseases, Capital Medical University, Beijing, China; ^4^Department of Radiology, Beijing Anzhen Hospital, Capital Medical University, Beijing, China; ^5^Cardiovascular Research Centre, Royal Brompton Hospital, London, United Kingdom; ^6^National Heart and Lung Institute, Imperial College London, London, United Kingdom

**Keywords:** dilated cardiomyopathy, cardiac magnetic resonance imaging, left ventricular global function index, association analysis, clinical outcomes

## Abstract

**Purpose:** Left ventricular global function index (LVGFI) assessed using cardiac magnetic resonance (CMR) seems promising in the prediction of clinical outcomes. However, the role of the LVGFI is uncertain in patients with heart failure (HF) with dilated cardiomyopathy (DCM). To describe the association of LVGFI and outcomes in patients with DCM, it was hypothesized that LVGFI is associated with decreased major adverse cardiac events (MACEs) in patients with DCM.

**Materials and Methods:** This prospective cohort study was conducted from January 2015 to April 2020 in consecutive patients with DCM who underwent CMR. The association between outcomes and LVGFI was assessed using a multivariable model adjusted with confounders. LVGFI was the primary exposure variable. The long-term outcome was a composite endpoint, including death or heart transplantation.

**Results:** A total of 334 patients (mean age: 55 years) were included in this study. The average of CMR-LVGFI was 16.53%. Over a median follow-up of 565 days, 43 patients reached the composite endpoint. Kaplan–Meier analysis revealed that patients with LVGFI lower than the cutoff values (15.73%) had a higher estimated cumulative incidence of the endpoint compared to those with LVGFI higher than the cutoff values (*P* = 0.0021). The hazard of MACEs decreased by 38% for each 1 SD increase in LVGFI (hazard ratio 0.62[95%CI 0.43–0.91]) and after adjustment by 46% (HR 0.54 [95%CI 0.32–0.89]). The association was consistent across subgroup analyses.

**Conclusion:** In this study, an increase in CMR-LVGFI was associated with decreasing the long-term risk of MACEs with DCM after adjustment for traditional confounders.

## Introduction

The most common causes of heart failure (HF) include ischemia dilated cardiomyopathy (IDCM) and non-ischemia dilated cardiomyopathy (NIDCM) (1). Both DCMs involve left ventricular (LV) dilation and contractile dysfunction, referring to cardiac remodeling underlying the morphological substrate of the clinical syndrome of HF, and are associated with adverse clinical outcomes in pathological conditions (2). A previous observational study (3) showed eccentric hypertrophy, in which myocardial mass was increased with cavity enlargement in 94% of the patients with DCM. Thus, cardiac remodeling patterns differ in LV volume, mass, and function in patients with HF. The increased LV mass is the same as the increased LV volume and depressed LV systolic function (ventricles exhibit eccentric hypertrophy), which is crucial for prognosis (4). Thus, the comprehensive evaluation of cardiac performance requires a combination of the structure and function measurement.

Numerous studies have demonstrated that the measurement of LV ejection fraction (LVEF) as a marker of global systolic myocardial function is a robust predictor of morbidity and mortality in patients with DCM (5, 6). However, LVEF does not account for the correlation between LV mass and dimension, and hence, it lacks the comprehensive evaluation of cardiac performance (7).

CMR imaging is the gold standard for evaluating cardiac function and morphology and plays a central role in the diagnosis of HF, the assessment of prognosis, and monitoring of therapy (8, 9). Recently, a new marker of LV global function index (LVGFI) from CMR that comprehensively incorporates LV volume and mass, as well as global systolic function, has been introduced to assess cardiac performance (7). In previous studies, LVGFI is a new and promising parameter that can predict adverse outcomes in healthy individuals (10). It is determined by CMR after STEMI, rendering it a robust predictor of MACEs at long-term follow-up of 3.1 years (11). However, the role of the LVGFI is unknown in patients with HF due to DCM. Herein, we hypothesized that a high LVGFI is associated with a decrease in MACEs in patients with DCM.

## Materials and Methods

This study followed the Strengthening the Reporting of Observational Studies in Epidemiology (STROBE) guidelines (12).

### Study Population and Design

Patients with stage C or D HF were identified from January 1, 2015 to April 30, 2020 and enrolled non-selectively and consecutively in this prospective observational cohort study at the Department of Heart Failure Program at Anzhen Hospital (Capital Medical University, Beijing, China). The current study enrolled 493 patients with DCM according to the World Health Organization/International Society guidelines and the Federation of Cardiology (13). According to the inclusion criteria of the study, patients with LVEF <40%, and LV end-diastolic diameter (LVEDD) >60 mm on echocardiography were enrolled. On the other hand, patients with congenital heart disease, infiltrative cardiomyopathy, valvular heart disease, acute myocardial infarction (MI) within 1 month, or a history of CRT and ICD implantation were excluded from this study. Supplementary Table 1 provides the complete list of epidemiological, clinical, biological variables, electrocardiogram, and imaging variables recorded for each patient.

This study protocol was approved by the Human Subjects Review Committee at Anzhen Hospital (Approval No. 2013007X). The patient's identity remained anonymous, and the requirement for informed consent was waived due to the observational design of this study, as reported elsewhere (14).

### CMR Protocol and Variables Definition

CMR acquisition was performed on a 3.0 T scanner (Magnetom Verio; Siemens AG Healthcare, Erlangen, Germany or Discovery™ MR750w, General Electric Healthcare, Waukesha, WI, USA) with retrospective electrocardiogram gating and 32-channel phased-array coil. The steady-state free precession breath-hold cine images with 25 reconstructed phases were acquired. The detailed scanning parameters of cine images have been described previously (15).

**Figure 1:**
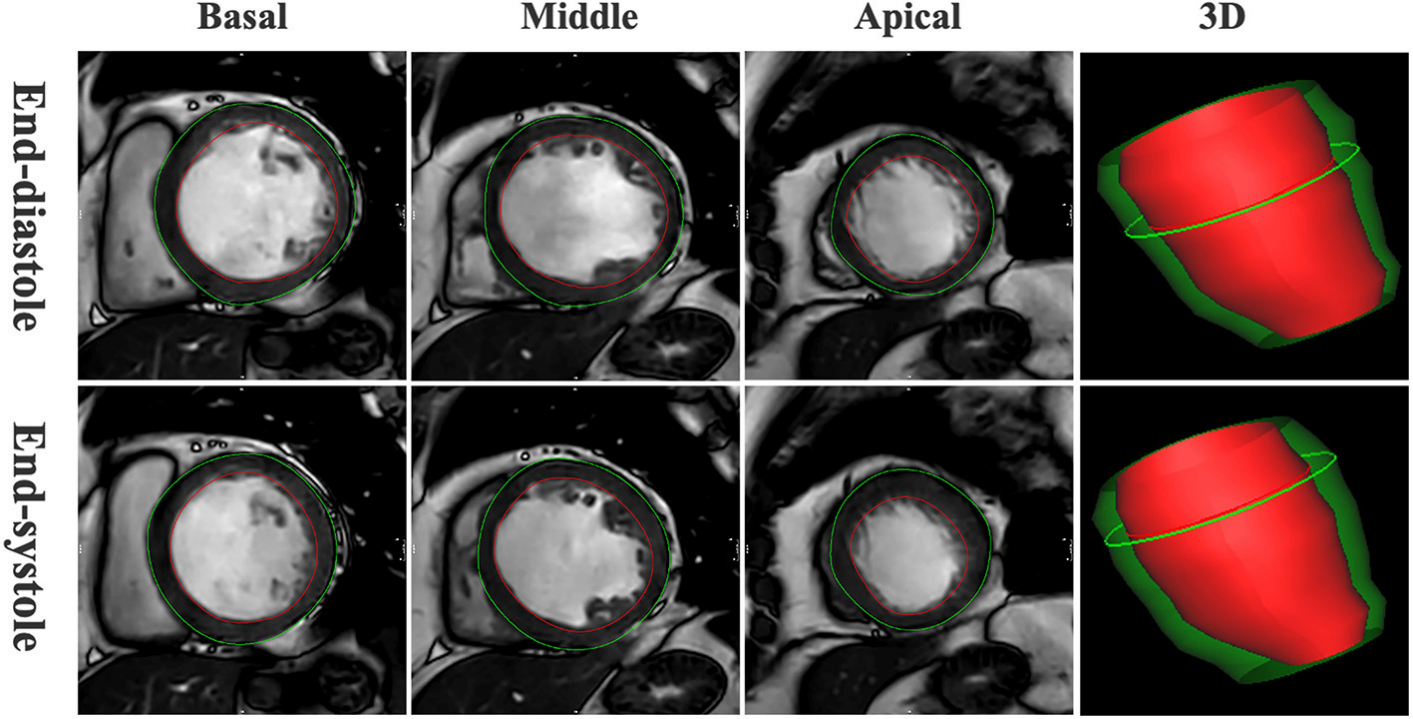
Representative example of left ventricular global function index (LVGFI) in a patient with dilated cardiomyopathy. Endocardial (red) and epicardial (green) borders were drawn on the short-axis cine images from basal segment to apical segment and then three-dimensional (3D) model of the left ventricle in end-diastole and end-systole were generated automatically.

One experienced and independent radiologist blinded to clinical data gathered the CMR readings for all standard cardiac parameters and LVGFI. The LVGFI was the primary exposure variable in this study and acquired by drawing endocardial and epicardial borders on the short-axis cine images from basal slice to apical segment (Figure 1). It was defined according to the following formula for each subject:


$$\begin{array}{l} \text{LVGFI}=\left(\text{LVSV}/\text{LVGV}\right)\;\times \;100\text{\%} \end{array}$$

where the LV stroke volume (LVSV) was calculated by LVEDV-LVESV, and the LV global volume (LVGV) was defined as the sum of the mean LV cavity volume (LVEDV+LVESV)/2 and the myocardium volume. The LV myocardial volume was calculated as the LV myocardial mass divided by the specific myocardial density (1.05 g/mL). Further details regarding CMR data assessment are described previously (15).

### Covariates

The selection of covariates is based on our previous studies (1, 9, 15), and those examining the risk factors for HF (16). Laboratory (17) and electrocardiogram (18) examinations provided data on hemoglobin (HgB), white blood cell counts (WBC), ion concentration, renal function, the level of brain natriuretic peptide (BNP), atrial fibrillation (AF) (19), and LBBB.

### Follow-Up and Study Endpoints

The attending physician of the patients had to fill out clinical information, and the biochemical and imaging data were obtained using the Empower DataWeb data collection management system (X&Y Solutions, Inc., Boston, MA, USA). The patients were followed up at a 3-month interval with the clinical outpatient visit or by contact with the patient's family members. The status of the events was updated on the Empower DataWeb each time.

The main outcome was the composite of major adverse cardiac events (MACEs), including cardiovascular death and cardiac transplantation. Cardiovascular death was defined as HF, fatal MI, sudden death, and ischemic or hemorrhagic stroke. The causes of death were based on the physician's judgment. The patients were followed up until the beginning of May 2020.

### Statistical Analysis

All statistical analyses were performed using EmpowerStats (www.empowerstats.com, X&Y Solutions, Inc. Boston MA) and R software version 3.6.1 (http://www.R-project.org). The two-side alpha level was set at 0.05. Continuous variables are presented as mean ± SD or medians and interquartile ranges (IQRs), while categorical variables are presented as frequencies and percentages. Box-Cox transformation was used to assess the skewed distribution variable. We used multiple imputations based on five replications and a chained equation approach method in the R approach to account for missing data. We also performed sensitivity analyses to compare the distributions of variables with missing data at observed complete case data and the pooling datasets with variables from multiple imputations.

Firstly, generalized additive models (GAMs) were used to identify the LVGFI and outcome correlations after adjusting the most covariates because LVGFI was a continuous variable (Supplementary Figure 1). GAM also assessed and explored the linear correlation between MACEs and confounders (Supplementary Figure 2) to fit the best model. Secondly, univariate Cox models were used to evaluate the association between each significant variable and MACEs in patients with DCM within 5 years of follow-up. Thirdly, multivariate Cox models were used to examine whether CMR-LVGFI had an independent effect on MACEs in patients with DCM within follow-up. When added to this model, the covariates altered the effect estimate of >10%, and covariates of known clinical importance were adjusted while omitting the collinear variables (20). Finally, the subgroup analysis deduced whether potential risk factors had an impact on the results. The interactions among the subgroups were examined using the multivariate-adjusted Cox model.

Time-dependent receiver operating characteristic curves (ROCs) were plotted to set the optimal cutoff of CMR-LVGFI for the primary composite endpoint based on the Youden index. The optimal cutoff values of LVGFI for the analyzed events were based on the highest sum of sensitivity and specificity. The event-free curves were based on Kaplan–Meier analysis stratified by LVGFI optimal cutoffs and compared using the log-rank test.

## Results

**Figure 2:**
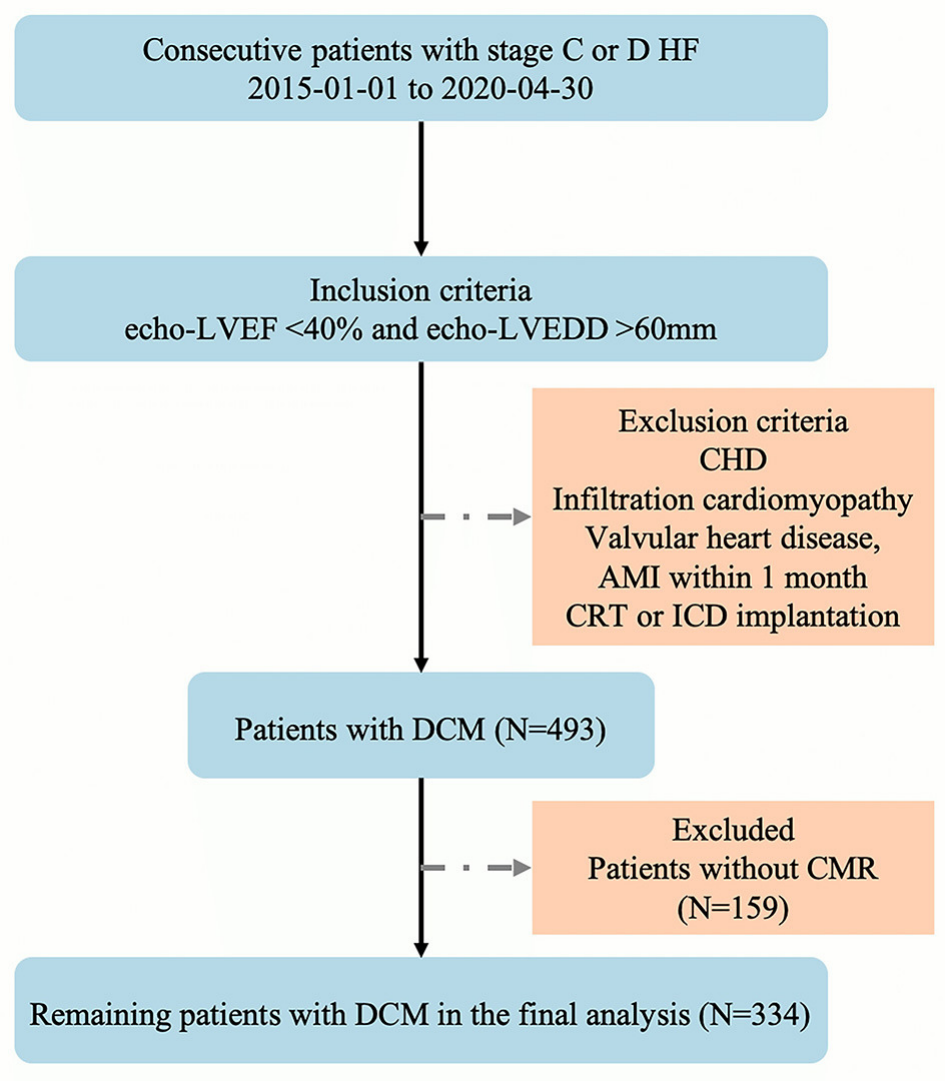
Flow chart of the observational study.

Between January 1, 2015 and April 30, 2020, a total of 493 patients with stage C or D HF who met the above inclusion criteria were enrolled in this study. However, 159 patients did not complete the CMR examination, and the final analysis included 334 patients (Figure 2) with DCM with stage C or D HF. The mean age of the patients was 55 ± 13 years, and 76.15% were males. In approximately one-third of patients, HF is caused by ischemic cardiomyopathy. Among the patients in the cohort study, 14% showed AF or LBBB on EKG, the average QRS interval was 119.95 ± 30.70 ms, and the mean LVEF and RVEF on CMR was 23.21 ± 10.41% and 33.62 ± 14.87%, respectively. The median BNP level was 263.50 pg/mL, as determined in a central laboratory. Furthermore, the concomitant guideline-based medical therapy was well used in the cohort study: 91.57% of the cohort was prescribed either ACEI or ARB or ARNI, 95.83% was prescribed a β-blocker, and 89.58% was prescribed an aldosterone antagonist (Table 1).

**Table 1:** Baseline characteristics of patients with HF.

**Mean± SD /**	**All patients**
**Median (interquartile ranges)**	**(*n* = 334)**
Age, mean (SD) (years)	55.17 ± 12.73
Male (%)	249 (76.15%)
BMI, mean (SD) (kg/m^2^)	25.96 ± 4.90
Ischemia cardiomyopathy (%)	97 (29.66%)
Diabetes mellitus (%)	85 (26.23%)
CKD (%)	9 (2.78%)
AF on EKG	45 (14.06%)
LBBB on EKG (%)	47 (14.73%)
RBBB on EKG (%)	15 (4.70%)
Intraventricular block on EKG (%)	24 (7.50%)
QRS duration mean (SD) (ms)	119.95 ± 30.70
SBP (mmHg)	117.99 ± 16.75
SBP ≥130 mmHg (%)	91 (28.44%)
HR (bpm)	78.49 ± 15.12
Alanine aminotransferase (ALT) (U/L)	25.00 (17.00–39.25)
Aspartate aminotransferase (AST) (U/L)	25.00 (19.00–30.00)
Creatinine mean (SD) (mmol/L)	84.47 ± 24.10
Na+ (SD) (mmol/L)	139.51 ± 2.94
Cl- (SD) (mmol/L)	104.97 ± 52.23
hs-CRP mean (SD) (mmol/L)	1.66 (0.61–4.67)
Homocysteine (Hcy) (umol/L)	15.10 (11.75–21.55)
WBC (G/L)	7.23 ± 2.11
HgB (G/L)	145.77 ± 22.37
Platelets (PLT) (G/L)	206.99 ± 65.76
BNP (pg/ml)	263.50 (125.50–547.50)
CMR-LVEDV (ml)	270.66 ± 103.69
CMR-LVESV (ml)	213.24 ± 98.24
CMR-LVEF (%)	23.21 ± 10.41
CMR-LVMASS (g)	142.92 ± 46.37
CMR- LVGFI (%)	16.53 ± 7.64
CMR-RVEDV (ml)	129.69 ± 47.10
CMR-RVESV (ml)	88.55 ± 44.22
CMR-RVEF (%)	33.62 ± 14.87
CMR-LV-LGE (%)	159 (47.89%)
**Medication**	
ACEI/ARB/ARNI (%)	304 (91.02%)
Beta-blocker (%)	311 (93.11%)
Spironolactone (%)	289 (86.52%)

*BMI, body mass index; CKD, chronic kidney disease; SBP, systolic blood pressure; hs-CRP, C-reactive protein; LBBB, left bundle branch block; RBBB, right bundle branch block; CMR, cardiac magnetic resonance; LVEDV, left ventricular end-diastolic volume; LVESV, left ventricular end-systolic volume; LVEF, left ventricular ejection fraction; LVMASS, left ventricular mass; RVEDV, right ventricular end-diastolic volume; RVESV, right ventricular end-systolic volume; RVEF, right ventricular ejection fraction; LVLGE, left ventricular late gadolinium enhancement; ACEI, angiotensin-converting enzyme inhibitor; ARB, angiotensin II receptor blocker; ARNI, angiotensin receptor neprilysin inhibitor; SD, standard deviation*.

### CMR-LVGFI Levels and the Risk of MACEs in Patients With DCM

A total of 43 patients developed MACEs in the median follow-up period of 565 days, consisting of 41 patient deaths and two heart transplantation patients. In the present study, we established a linear inverse correlation between LVGFI and Log RR for MACEs after adjusting for sex, age, BMI, ischemia cardiomyopathy, LBBB on EKG, QRS duration, RBBB on EKG, intraventricular block on EKG, AF on EKG; Na+, Cl–, BNP, Hb, creatinine, WBC, LV-LGE, RVEDV, and RVEF, respectively (Supplementary Figure 1).

The results of univariate analyses of MACEs are summarized in Table 2. The univariate analyses showed that ischemia cardiomyopathy, QRS duration, AF on EKG, creatinine, BNP, Na+, Cl-, CMR-LVEDV, CMR-LVESV, CMR-LVMASS, and CMR-LVGFI was associated with a significant increase in the incidence of MACEs. Next, we performed a multivariate Cox regression analysis to further explore CMR-LVGFI as a prognostic marker. In the multivariable analysis shown in Table 3, CMR-LVGFI level is negatively associated with the risk of MACEs in Model I (hazard ratio adjusted (HRadj) 0.51 per 1 SD increment, 95% confidence interval (CI): 0.33–0.77; *P* = 0.0013) after adjusting for age, sex, and BMI. This was also true in Model II (HRadj 0.54 per 1 SD increment, 95% CI: 0.35–0.82; *P* = 0.0044) after adjusting for sex, age, BMI, and ischemia cardiomyopathy, LBBB on EKG, QRS duration, RBBB on EKG, intraventricular block on EKG, and AF on EKG; Model III (HRadj 0.63 per 1 SD increment, 95% CI: 0.42–0.96; *P* = 0.0302) after adjusting for sex, age, BMI, Na+, Cl–, BNP, Hb, creatinine, white blood cell; Model IV (HRadj 0.93 per 1 SD increment, 95% CI: 0.87–0.98; *P* = 0.0126), after adjusting for sex, age, BMI, LV-LGE, RVEDV, RVEF; Model V (HRadj 0.54 per 1 SD increment, 95% CI: 0.32–0.89; *P* = 0.01654) was obtained after adjusting for ischemia cardiomyopathy, LBBB on EKG, QRS duration, RBBB on EKG, intraventricular block on EKG, AF on EKG, Na+, Cl–, BNP, Hb, creatinine, white blood cell, LV-LGE, RVEDV, and RVEF.

**Table 2:** Univariate Cox analysis for MACEs in patients with HF within the median follow-up period of 565 days.

	**HR**	**95%CI**	***P*-value**
Age (years)	1.03	1.01, 1.06	0.0153
Male	1.30	0.60, 2.80	0.5077
BMI (kg/m^2^)	0.95	0.88, 1.02	0.1691
Ischemia cardiomyopathy (%)	1.77	0.96, 3.27	0.00674
Diabetes mellitus (%)	1.46	0.78, 2.74	0.2359
QRS duration (mm)	1.01	1.00, 1.02	0.0048
AF on EKG	2.40	1.23, 4.69	0.0103
LBBB on EKG (%)	1.61	0.79, 3.28	0.1908
RBBB on EKG (%)	0.80	0.19, 3.31	0.7538
Intraventricular block (%)	1.82	0.77, 4.34	0.1733
SBP (mmHg)	0.98	0.96, 1.00	0.0657
SBP≥130 mmHg (%)	0.66	0.31, 1.43	0.2954
ALT (U/L)	1.00	0.99, 1.01	0.9741
AST (U/L)	1.00	0.99, 1.01	0.9742
Creatinine (μmol/L)	1.01	1.00, 1.02	0.0092
Na+ (SD) (mmol/L)	0.93	0.86, 0.99	0.0358
Cl- (SD) (mmol/L)	1.00	1.00, 1.01	0.0004
hs-CRP (mmol/L)	1.02	0.98, 1.06	0.2893
Hcy (umol/L)	0.99	0.98, 1.01	0.3119
WBC (G/L)	0.92	0.78, 1.08	0.3108
HgB (G/L)	1.00	0.99, 1.01	0.4533
PLT (G/L)	1.00	0.99, 1.00	0.2146
BNP (Box-Cox transform) (pg/ml)	1.36	1.15, 1.59	0.0002
CMR-LVEDV (ml)	1.00	1.00, 1.01	<0.0001
CMR-LVESV (ml)	1.00	1.00, 1.01	<0.0001
CMR-LVEF %	0.95	0.92, 0.99	0.0074
CMR-LVMASS (g)	1.01	1.00, 1.01	0.0090
CMR-LVGFI (per 1% increase)	0.94	0.89, 0.99	0.0146
CMR-LVGFI (per 1 SD increase)	0.62	0.43, 0.91	0.0146
CMR-RVEDV (ml)	1.01	1.00, 1.01	0.0555
CMR-RVESV (ml)	1.01	1.00, 1.01	0.1429
CMR-RVEF %	1.00	0.98, 1.02	0.8097
CMR-LV-LGE	1.02	1.01, 1.04	0.0040
**Medication**	
ACEI/ARB/ARNI (%)	0.62	0.22, 1.75	0.3683
Beta-blocker (%)	0.61	0.19, 1.98	0.4126
Spironolactone (%)	2.48	0.60, 10.27	0.2113

*BNP has done the Box-Cox transformation that Box-Cox transform formula is (BNP ^∧^0.1418−1)/0.1418. HR, Hazard ratio; CI, Confidence interval*.

**Table 3:** Multivariate Cox analysis with CMR- LVGFI for MACEs in patients with HF within the median follow-up period of 565 days.

**Exposure**	**Model I**	**Model II**	**Model III^#^**	**Model IV**	**Model V^#^**
	**(*n* = 315)**	**(*n* = 299)**	**(*n* = 334)**	**(*n* = 312)**	**(*n* = 334)**
LVGFI, per 1% increase	0.91 (0.87, 0.97) 0.0013	0.92 (0.87, 0.97) 0.0044	0.94 (0.89, 0.99) 0.0249	0.93 (0.87, 0.98) 0.0126	0.92 (0.86, 0.99) 0.01639
LVGFI, per 1 SD increase	0.51 (0.33, 0.77) 0.0013	0.54 (0.35, 0.82) 0.0044	0.63 (0.42, 0.96) 0.0302	0.56 (0.35, 0.88) 0.0126	0.54 (0.32, 0.89) 0.01654
**LVGFI group**					
<15.73	1.0	1.0	1.0	1.0	1.0
≥15.73	0.27 (0.13, 0.60) 0.0012	0.29 (0.12, 0.68) 0.0043	0.32 (0.14, 0.72) 0.0059	0.28 (0.12, 0.67) 0.0044	0.26 (0.10, 0.68) 0.00665
**LVGFI group**					
T1	1.0	1.0	1.0	1.0	1.0
T2	0.90 (0.46, 1.74) 0.7436	1.00 (0.50, 1.99) 0.9945	1.16 (0.59, 2.29) 0.6629	1.08 (0.54, 2.19) 0.8246	1.37 (0.64, 2.94) 0.42277
T3	0.26 (0.10, 0.69) 0.0073	0.23 (0.07, 0.71) 0.0102	0.32 (0.11, 0.89) 0.0289	0.26 (0.08, 0.85) 0.0251	0.19 (0.05, 0.76) 0.01885

*Model I adjust for: sex, age, BMI*.

*Model II adjust for: sex, age, BMI, Ischemia cardiomyopathy, LBBB on EKG, QRS duration, RBBB on EKG, Intraventricular block on EKG, AF on EKG*.

*Model III adjust for: sex, age, BMI, Na+, Cl–, BNP (BOXCOX transformation), Hb, Creatinine, White blood cell*.

*Model IV adjust for: sex, age, BMI, LV-LGE, RVEDV, RVEF, while omitting collinear variables*.

*Model V adjust for: sex, age, BMI, Ischemia cardiomyopathy, LBBB on EKG, QRS duration, RBBB on EKG, Intraventricular block on EKG, AF on EKG; Na+, Cl-, BNP (BOXCOX transformation), Hb, Creatinine, White blood cell; LV-LGE, RVEDV, RVEF*.

*LVGFI was dichotomized according to optimized cutoff values, which was calculated by ROC analysis, and LVGFI was categorized in tertile*.

# *Data on the BNP, age, sex, BMI, creatinine, Na+, Cl–, WBC and hemoglobin were missing for 100 (29.9%), 8 (2.4%), 7 (2.1%), 18 (5.4%), 31 (9.3%), 37 (11.1%), 38 (11.4%), 43 (12.9%) and 43 (12.9%) patients, respectively. Multiple imputations were used to account for missing data in Model III and Model V*.

**Figure 3:**
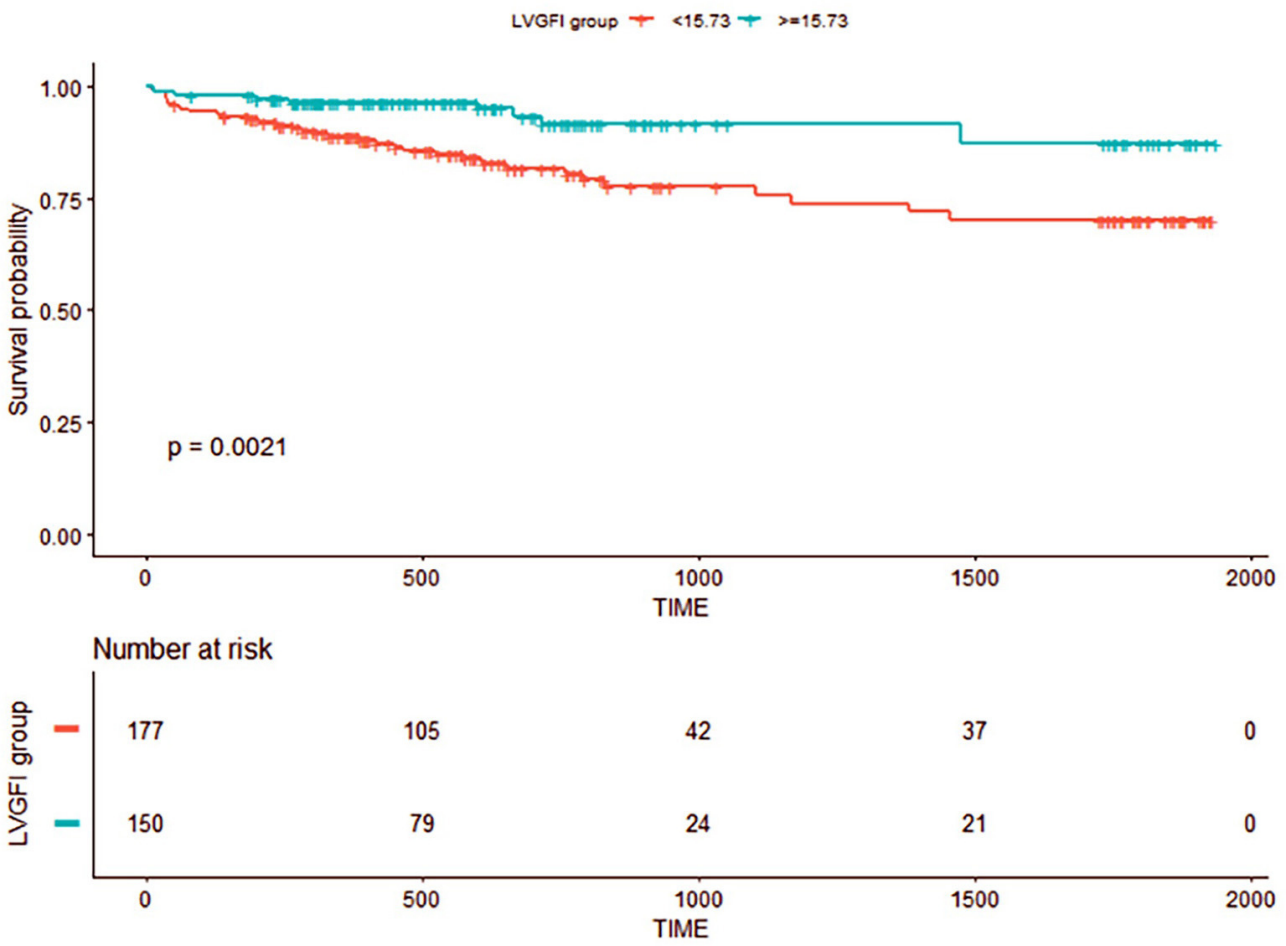
Kaplan–Meier event-free survival curve. Numbers that do not add up to 334 are attributed to the missing data for LVGFI.

The time-dependent ROC curves that we used for bootstrap resampling (times = 500) were constructed to determine the optimal cutoff of CMR-LVGFI for the primary composite with a 15.73% endpoint (Supplementary Figure 3). The dichotomized CMR-LVGFI value by 15.73 was used in Kaplan–Meier survival analysis (Figure 3), which showed a significant difference among patients stratified by the LVGFI value by 15.73%. A decreased LVGFI (LVGFI <15.73%) was associated with a reduced MACE-free survival (log-rank *P* = 0.0021).

### Subgroup Analysis Between CMR-LVGFI Levels and MACEs in Patients With Stage C or D HF With DCM

To evaluate the potential influence of other factors, the subgroup analysis was conducted after stratifying the patients by age, BMI, and SBP, sex, diabetes mellitus, LBBB on EKG, ischemia cardiomyopathy, AF on EKG, and CMR-LV-LGE (Table 4). Notably, all subgroups demonstrated a similar overall correlation between CMR-LVGFI levels and the risk of MACEs in patients with stage C or D HF with DCM.

**Table 4:** Effects of CMR- LVGFI on MACEs in patients with HF in each subgroup by multivariable Cox model within the median follow-up period of 565 days.

**CMRI- LVGFI**	HR (95% CI)^*^	***P*-value**	***P* for interaction**
Age (n, %)			0.7131
≤ 51 years (108, 33.13%)	0.96 (0.86, 1.07)	0.4937	
52-61 years (108, 33.13%)	0.92 (0.84, 1.01)	0.0730	
≥62 years (110, 33.74%)	0.90 (0.83, 0.98)	0.0178	
Sex (n, %)			0.2183
Female (78, 23.85%)	0.97 (0.88, 1.07)	0.5976	
Male (249, 76.15%)	0.91 (0.85, 0.97)	0.0035	
BMI (n, %)			0.7319
≤ 24 (105, 33.23%)	0.94 (0.87, 1.02)	0.1631	
24–26 (105, 33.23%)	0.90 (0.82, 0.98)	0.0215	
≥26 (106, 33.54%)	0.93 (0.81, 1.06)	0.2866	
SBP≥130 mmHg (n, %)			0.8546
No (229, 71.56%)	0.93 (0.87, 0.98)	0.0125	
Yes (91, 28.44%)	0.91 (0.79, 1.05)	0.2000	
Diabetes mellitus (n, %)			0.3678
No (239, 71.56%)	0.94 (0.88, 1.00)	0.0597	
Yes (85, 25.45%)	0.89 (0.80, 0.98)	0.0243	
Ischemic cardiomyopathy (n, %)			0.3767
No (230, 68.86%)	0.95 (0.88, 1.02)	0.1594	
Yes (97, 29.04%)	0.90 (0.83, 0.98)	0.0121	
AF on EKG (n, %)			0.6913
No (275, 82.34%)	0.92 (0.86, 0.98)	0.0074	
Yes (45, 13.47%)	0.94 (0.84, 1.06)	0.3442	
LBBB on EKG (n, %)			0.6407
No (272, 81.44%)	0.92 (0.86, 0.98)	0.0093	
Yes (47, 14.07%)	0.95 (0.85, 1.05)	0.3064	
CMR-LV-LGE (n, %)			0.1327
No (173, 51.8%)	0.98 (0.90, 1.07)	0.6205	
Yes (159, 47.6%)	0.90 (0.83, 0.96)	0.0029	

* *Adjusted for age, sex, BMI, SBP, Diabetes mellitus, LBBB on EKG, Ischemia cardiomyopathy, AF on EKG, and CMRI-LV-LGE except for the subgroup variable*.

## Discussion

This large prospective cohort study investigated the association between CMR-derived LVGFI and outcomes in patients with stage C or D HF with DCM in China. The current findings demonstrated that the long-term risk of the composite endpoint in patients with DCM over a median follow-up period of 565 days was significantly negatively associated with CMR-LVGFI integrating LV structure with global function. For every 1 SD increase in CMR-LVGFI, a HR of the composite endpoint after adjusting the clinical, laboratory, electrocardiogram, and CMR routine variables decreased by 46%, which was evident in all the subgroups after adjustments.

The changes in LV structure and geometry, termed cardiac remodeling, involve chamber dilation and/or hypertrophy. The patterns of LV remodeling were classified as concentric hypertrophy and remodeling, physiological hypertrophy, and eccentric hypertrophy and remodeling. Fifty years ago, Linzbach (21) proposed the concept of LV structural remodeling and developed a classification system for LV remodeling patterns. In 1965, Grant et al. (3) defined eccentric hypertrophy as an increased myocardial mass with LV chamber enlargement. Based on the M-mode echocardiography data from 4,975 participants in the Framingham Heart Study, Savage et al. (22) described two subtypes of LV eccentric hypertrophy, referred to as “eccentric dilated” and “eccentric non-dilated.” Typically, patients with depressed systolic function exhibit LV enlargement, accompanied by eccentric hypertrophy. A previous observational study showed that the most common pattern of LV structural remodeling is eccentric hypertrophy, in which myocardial mass is increased with chamber dilatation; this phenomenon was detected in 94% of the patients with DCM (23). Thus, those with a dilated ventricle might have eccentric hypertrophy and low EF. Additionally, LV remodeling is accompanied by decreased cardiac function and is also associated with adverse outcomes. Furthermore, this remodeling pattern has been emphasized by the HF guidelines (24) and has become the target of HF treatment (25, 26). However, the relative importance of structural vs. functional abnormalities in a failing heart is currently under intensive research focus. The vast majority of studies emphasized LVEF as a maker of global systolic myocardial function, deeming it a robust predictor of morbidity and mortality in patients with DCM (5). However, LVEF only focuses on the cardiac functional status and does not account for the LV geometry and hypertrophy. Importantly, a comprehensive definition of cardiac performance requires more than a single measure of structure or function. LVGFI is a combination of structural and functional parameters and provides a rational and comprehensive description of cardiac performance.

LVGFI was initially introduced by Mewton et al. (10). The assessment of LVGFI incorporates elements of LV cavity size and mass, reflecting cardiac remodeling. Currently, it is a promising LV function index for screening, surveillance in individuals with CVD (cardiovascular disease), and risk-stratification in the general population (7, 10). Previous studies have shown that LVGFI by ECHO (7) and CMR (10) was independently associated with the subsequent development of HF, cardiovascular events, and a combined endpoint of all adverse events in community-dwelling individuals. Our results are consistent with these findings and could be extrapolated to patients with DCM. To the best of our knowledge, this is the first comprehensive study evaluating the correlation between CMR-LVGFI and future cardiovascular events in patients with DCM. Moreover, the study included patients with ischemic or non-ischemic cardiomyopathy. These associations persisted even after accounting for confounding and stratification factors, indicating that LVGFI is a reliable LV functional index because it reflects the comprehensive cardiac performance combining LV global systolic performance information with anatomical LV parameters. However, no significant differences were detected between the areas under the curve (AUC) obtained with LVGFI and LVEF on the MACEs (LVGFI ROC area: 0.653, LVEF ROC area: 0.659, *P* = 0.6) in our study (Supplementary Figure 4). Interestingly, three differences were noted between our and previous studies. Firstly, our study population comprised of patients with DCM, while that in the previous study was a normal community population. Secondly, the measurement method of LVGFI in our study was novel with reliable CMR imaging techniques, while that in the previous study was based on echocardiography. Finally, the primary outcome in our study was cardiovascular death and cardiac transplantation, while the primary endpoint in the previous study was incident symptomatic HF. Furthermore, from a large multicenter CMR study with patients of acute MI treated with percutaneous coronary intervention, LVGFI did not provide incremental prognostic information about LVEF (27).

Nevertheless, the present study has several limitations. This is a single-center study with a limited number of patients that might restrict the generalizability of these results. Based on prospective data collection and with adequate control of potential confounding factors, it is necessary to test the hypothesis in a large real-world cohort. Since the attending physician determined the cause of death, a subjective bias cannot be ruled out, which is similar to the current cohort study, unless systematic autopsy becomes the standard. Finally, the data of the BNP covariate was missing in 29.9% of the participants. However, statistical techniques employed multiple imputations to address the issue of missing data, and sensitivity analyses indicated that our assumptions regarding the patterns of missing data were reasonable (Supplementary Table 2).

## Conclusion

The LVGFI, integrating LV structure with global function, is independently associated with the long-term risk of MACEs in patients with stage C or D HF with DCM during follow-up. This suggested that LVGFI is a reliable LV functional and prognostic index because it reflects the cardiac performance at different degrees of structural LV remodeling.

## Data Availability Statement

The raw data supporting the conclusions of this article will be made available by the authors, without undue reservation.

## Ethics Statement

The studies involving human participants were reviewed and approved by the Human Subjects Review Committee at Anzhen Hospital (Approval No. 2013007X). Written informed consent to participate in this study was provided by the participants' legal guardian/next of kin.

## Author Contributions

LX and JD are the guarantors of integrity of the entire study. TL, JD, and LX are responsible for the study concepts and design. TL, ZZ, KB, YG, HW, RW, WL, SC, YL, and YS performed the experimental studies and prepared the first draft of the manuscript, which was critically revised by GY, DF, JD, and LX. TL, ZZ, KB, YG, and HW analyzed and interpreted the data. TL and ZZ performed statistical analysis and all the results were checked by JD and LX. All authors contributed to the article and approved the submitted version.

## Funding

This study was supported by a grant from the National Natural Science Foundation of China (U1908211), the National Key Research and Development Program of China (2016YFC1300300 and 2016YFC1301002), and the Capital's Funds for Health Improvement and Research Foundation of China (2020-1-1052). This study was also supported in part by the British Heart Foundation (TG/18/5/34111, PG/16/78/32402), the European Research Council Innovative Medicines Initiative (DRAGON, H2020-JTI-IMI2 101005122), the AI for Health Imaging Award (CHAIMELEON, H2020-SC1-FA-DTS-2019-1 952172), and the UK Research and Innovation Future Leaders Fellowship (MR/V023799/1).

## Conflict of Interest

The authors declare that the research was conducted in the absence of any commercial or financial relationships that could be construed as a potential conflict of interest.

## Publisher's Note

All claims expressed in this article are solely those of the authors and do not necessarily represent those of their affiliated organizations, or those of the publisher, the editors and the reviewers. Any product that may be evaluated in this article, or claim that may be made by its manufacturer, is not guaranteed or endorsed by the publisher.
