# Supplementary figures and images for: Association Between Left Ventricular Global Function Index and Outcomes in Patients With Dilated Cardiomyopathy
Source: Front Cardiovasc Med. 2021 Nov 16;8:751907. doi: 10.3389/fcvm.2021.751907 (PMC8635067; doi:10.3389/fcvm.2021.751907)

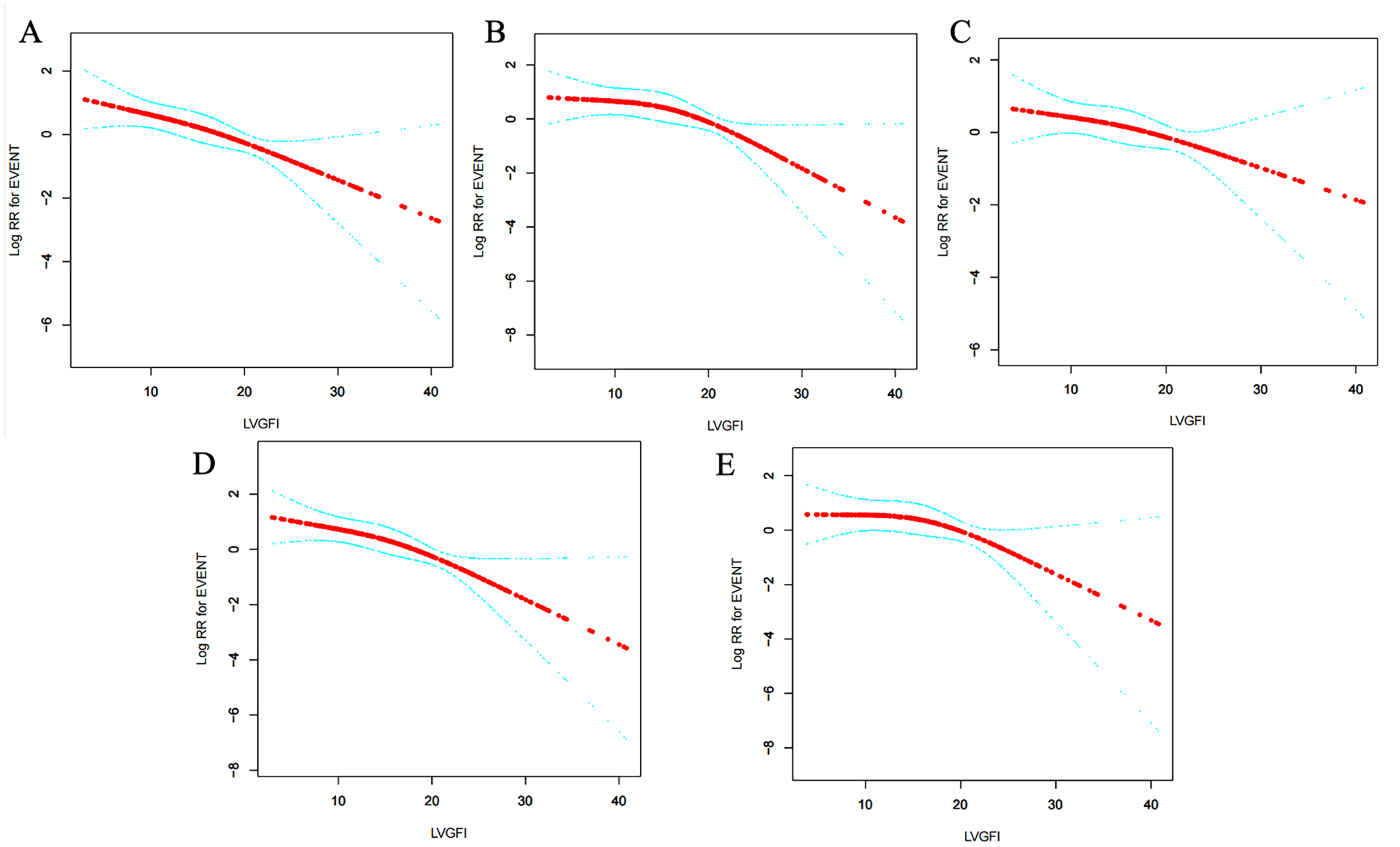

Supplement: Supplementary file 1 [file Image_1.TIF]

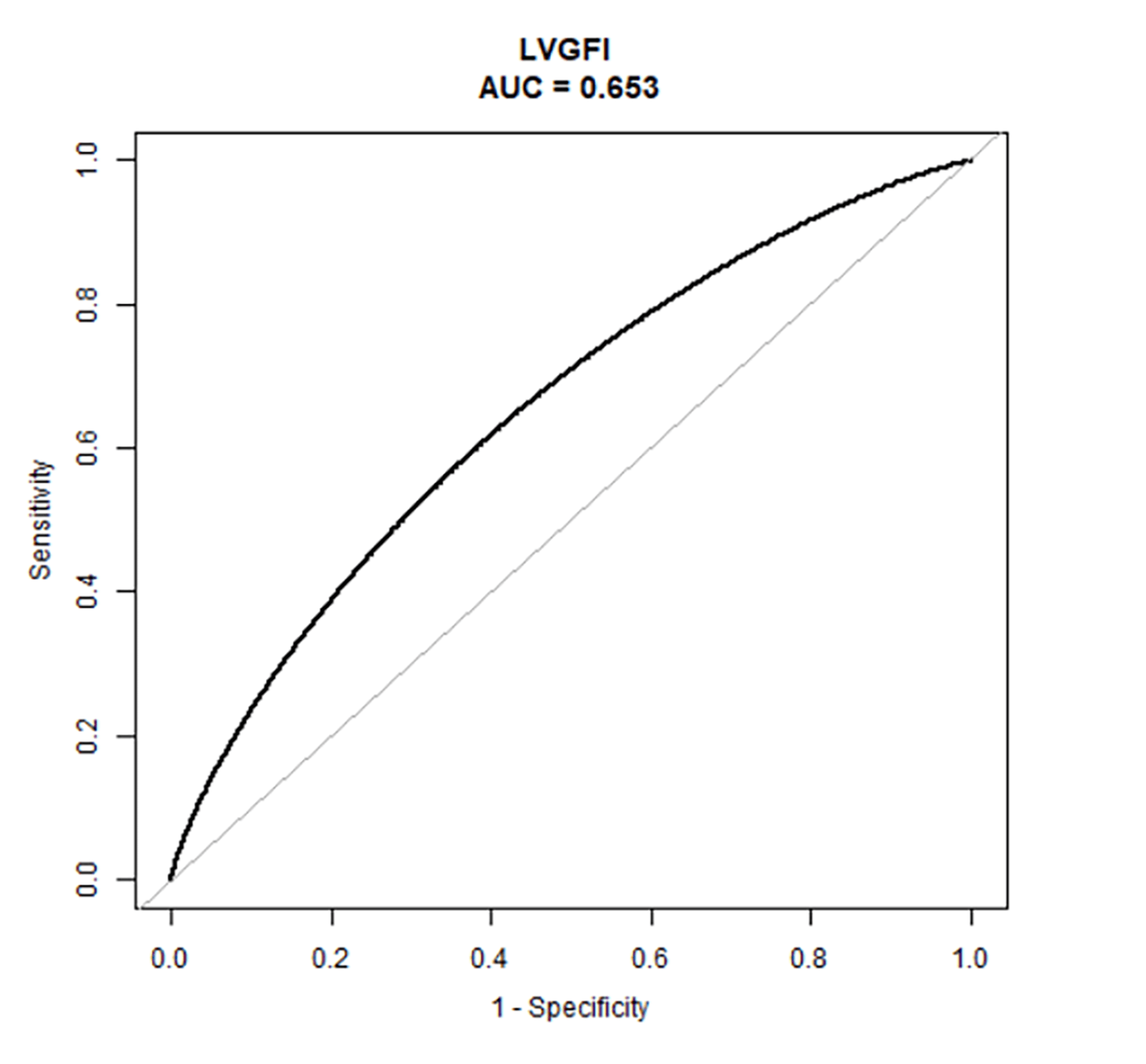

Supplement: Supplementary file 3 [file Image_3.TIF]

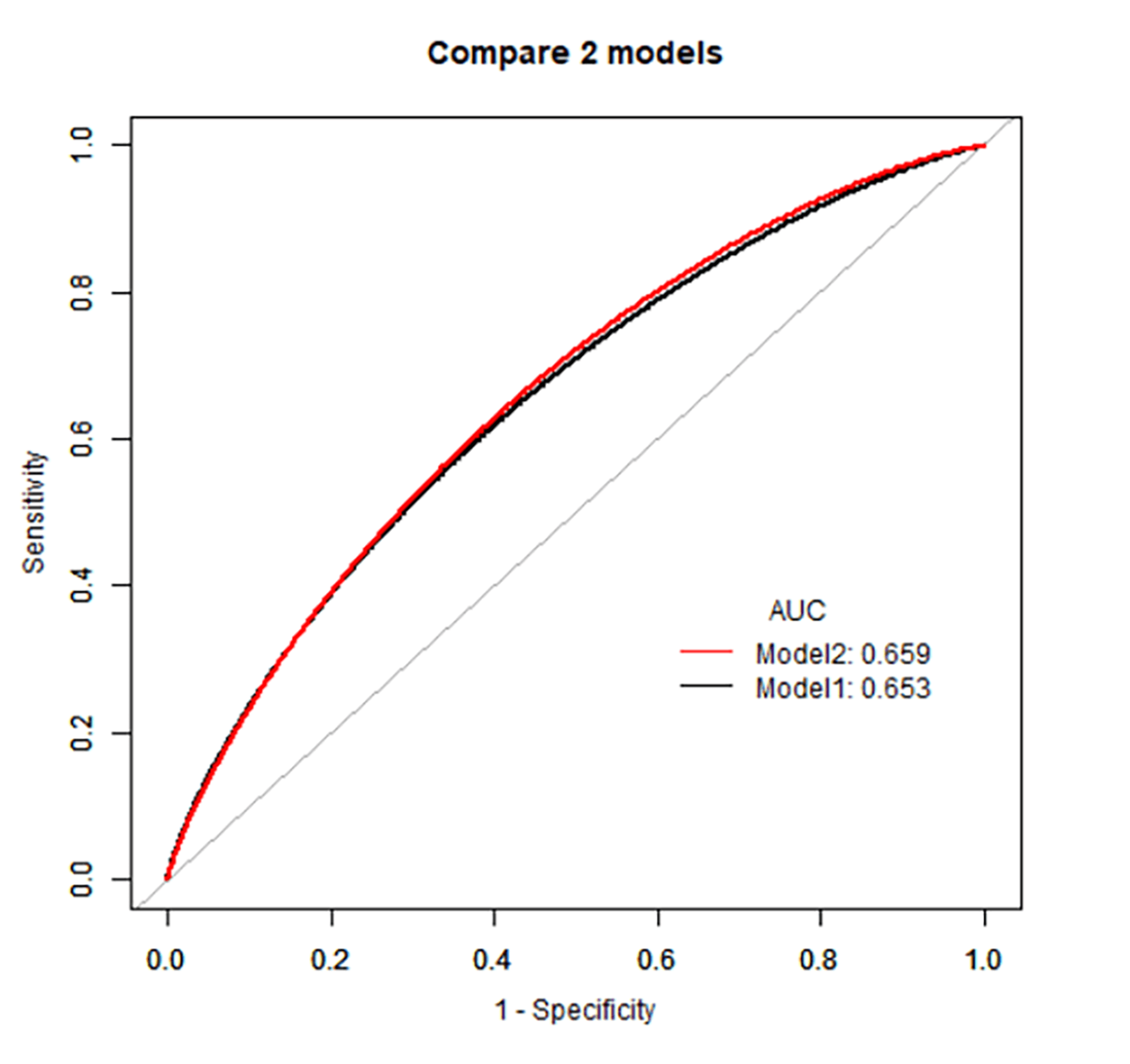

Supplement: Supplementary file 4 [file Image_4.TIF]
