# Supplementary material for: Association Between Left Ventricular Global Function Index and Outcomes in Patients With Dilated Cardiomyopathy
Source: Front Cardiovasc Med. 2021 Nov 16;8:751907. doi: 10.3389/fcvm.2021.751907 (PMC8635067; doi:10.3389/fcvm.2021.751907)
Supplement: Supplementary file 5 [file Data_Sheet_1.docx]

**Supplementary Tables**

**Table S1.** Complete list of collected variables.

| **Demographics** | **Clinical** | **Laboratory** | **Electrocardiogram** | **CMR imaging** | **Outcome and follow-up** |
| --- | --- | --- | --- | --- | --- |
| Age | Ischemia cardiomyopathy | Alanine aminotransferase | Heart rhythm | LVEDV | Cardiovascular death |
| Gender | Diabetes mellitus | Aspartate aminotransferase | LBBB | LVESV | Cardiac transplantation |
| weight | Chronic kidney disease | Creatinine | RBBB | LVEF |  |
| height | Blood pressure | Na+ | QRS duration | LVMASS |  |
|  | Heart rate | Cl- |  | LVGFI |  |
|  | Medication history | hs-CRP |  | RVEDV |  |
|  |  | Homocysteine |  | RVESV |  |
|  |  | White blood cell |  | RVEF |  |
|  |  | Hemoglobin |  | LV-LGE |  |
|  |  | Platelets |  |  |  |
|  |  | Brain natriuretic peptide |  |  |  |

**Table S2.** Sensitivity comparative analysis between patients with vs. without missing data

|  | Level/Unit | Number (%) with missing data | Complete case | Multiple imputation |
| --- | --- | --- | --- | --- |
| BNP (Box-Cox transform) (pg/ml) | Mean | 100 (29.9%) | 8.57 | 8.99 |
| Age (year) | Mean | 8 (2.4%) | 55 | 55 |
| Sex | % | 7 (2.1%) |  |  |
| BMI (kg / m2) | Mean | 18 (5.4%) | 26 | 26 |
| Creatinine(mmol/L) | Mean | 31 (9.3%) | 84.46 | 84.56 |
| Na+ (mmol/L) | Mean | 37 (11.1%) | 139.5 | 139.3 |
| Cl- (mmol/L) | Mean | 38 (11.4%) | 104.9 | 104.9 |
| WBC (mm) | Mean | 43 (12.9%) | 7.23 | 7.43 |
| Hemoglobin (HgB) (G/L) | Mean | 43 (12.9%) | 145.8 | 145.9 |

Nearly all variables of missing data were similar in patients with available data and patients with multiple imputation data.

**Note:** These tables are intended for publication as an online data supplement.
