# Supplementary material for: Association Between Left Ventricular Global Function Index and Outcomes in Patients With Dilated Cardiomyopathy
Source: Front Cardiovasc Med. 2021 Nov 16;8:751907. doi: 10.3389/fcvm.2021.751907 (PMC8635067; doi:10.3389/fcvm.2021.751907)
Supplement: Supplementary file 6 [file Data_Sheet_2.docx]

**Supplementary figure legends**

**Figure S1.** Adjusted generalized addition model for correlations between CMR-LVGFI and MACEs in patients with dilated cardiomyopathy. (A) Adjusting for sex, age and BMI; (B) Adjusting for sex, age BMI, ischemia cardiomyopathy, LBBB on EKG, QRS duration, RBBB on EKG, intraventricular block on EKG and AF on EKG; (C) Adjusting for sex, age, BMI, Na+, Cl-, BNP, Hb, Creatinine and WBC; (D) Adjusting for sex, age, BMI, LV-LGE, RVEDV and RVEF; (E) Adjusting for sex, age, BMI, ischemia cardiomyopathy, LBBB on EKG, QRS duration, RBBB on EKG, intraventricular block on EKG, AF on EKG, Na+, Cl-, BNP, Hb, Creatinine, WBC, LV-LGE, RVEDV and RVEF. MACEs: Major adverse cardiac events.

**Figure S2.** Generalized additive model for correlations between confounders and MACEs in patients with dilated cardiomyopathy. (A)BMI; (B)Age; (C)WBC; (D) Creatinine; (E)BNP; (F)Hb; (G) QRS duration; (H)RVEDV; (I) RVEF. MACEs: Major adverse cardiac events.

**Figure S3.**  ROC curves for the association of CMR-LVGFI and major adverse cardiac events. The analysis reveals that the cutoff LVGFI 15.73 (area under the curve: 0.653; sensitivity: 79.1%; specificity: 49.7%).

**Figure S4.** ROC curves of the predictive power of LVGFI and LVEF. The areas under curve represent the predictive power of LVGFI and LVEF with a non-adjusted Cox-regression model on the presence/absence of the major adverse cardiac events.

**Note:** These figures are intended for publication as an online data supplement.
